# Supplementary material for: A solution-processable and ultra-permeable conjugated microporous thermoset for selective hydrogen separation
Source: Nat Commun. 2020 Apr 2;11:1633. doi: 10.1038/s41467-020-15503-6 (PMC7118162; doi:10.1038/s41467-020-15503-6)
Supplement: Supplementary file 1 — Supplementary Information [file 41467_2020_15503_MOESM1_ESM.pdf]

A Solution-Processable and Ultra-Permeable Conjugated Microporous Thermoset for Selective Hydrogen Separation

Wei Liu[1,2,+], Shu-Dong Jiang[2,+], Youguo Yan[3,+], Wensen Wang[3], Jing Li[1], Kai Leng[1], Susilo Japip[2], Jiangtao Liu[2], Hai Xu[1], Yanpeng Liu[1], In-Hyeok Park[1], Yang Bao,[1] Wei Yu,[1] Michael D. Guiver[4,*], Sui Zhang[2,*], Kian Ping Loh[1,*]

[1]Department of Chemistry, National University of Singapore, 3 Science Drive 3, Singapore 117543, Singapore

[2] Department of Chemical and Biomolecular Engineering, National University of Singapore, 4 Engineering Drive 4, Singapore 117585, Singapore

[3]School of Materials Science and Engineering, China University of Petroleum, Qingdao 266580, Shandong, China.

[4]State Key Laboratory of Engines, Tianjin University, Tianjin 300072, China, & Collaborative Innovation Center of Chemical Science and Engineering (Tianjin), Tianjin 300072, China

[+]: These authors contributed equally to this work.

Correspondence should be addressed to: guiver@tju.edu.cn; chezhangsui@nus.edu.sg; chmlohkp@nus.edu.sg;

Table of contents

1. Material synthesis and preparation ······························4

1.1. Preparation of 3D, 2D and 1D conjugated microporous thermoset (CMT) 4

1.1.1. Synthesis of patterned 3D CMT ······························4

1.1.2. Preparation of 2D CMT on Si Wafer ······························4

1.1.3. Preparation of 1D CMT nanotubes ································5

1.1.4. Preparation of CMT ultrathin sheets ·····························5

2. Structural characterization of 3-TBTBP and CMT ··························6

2.1.Methods and instruments ··6

2.2. Crystallography data collection ···7

2.3. Crystal structure of 3-TBTBP ···8

2.4. X-ray photoelectron spectroscopy (XPS) spectra of 3-TBTBP and CMT 9

2.5. FTIR spectra of 3-TBTBP and CMT ···································10

2.6. Solid state ^{13}C NMR spectroscopy of 3-TBTBP and CMT ··········11

2.7. Elemental analyses of 3-TBTBP and CMT ····························11

2.8. SEM images of 3-TBTBP crystals and CMT ··························12

2.9. AFM image of a CMT film grown on Si wafer ························13

2.10. Raman spectrum of CMT film on Si wafer ···························13

2.11. SEM image of CMT coated Cu nanowires ····························14

2.12. Free volume diameter distribution in CMT ···························14

2.13. Optical properties of CMT film ···15

2.14. Optical microscope image of CMT sheets ·····························16

2.15. TEM image of CMT sheets ···16

2.16. XRD profile of CMT ···17

3. Gas permeation measurements ··18

3.1. Gas permeability and ideal selectivity of CMT membranes ·········19

3.2. Mixed H_2/CO_2 gas separation performance and CO_2 sorption isotherms of CMT membrane ···21

3.3. Comparison of gas separation performance of CMT with various reported membranes ··22

4. Molecular dynamics simulations ············ 24
4.1. Construction and validation of CMT model. ············ 24
4.2. MD simulation of gas separation ············ 26
4.2.1 Gas separation model construction ············ 26
4.2.2 MD simulation of gas separation process ············ 26
5. Crystallographic data of 3-TBTBP ············ 28
6. Supplementary References ············ 29

1. Material synthesis and preparation

1.1. Preparation of 3D, 2D and 1D conjugated microporous thermoset (CMT)

The thermosetting polymerization was conducted in a tube furnace with an Ar gas flow of 100 ml min^{-1}. In the tube furnace, precursors with various substrates were put in a jar equipped with a lid to minimize the disturbance of gas flow. Normally, samples were heated to 540 ℃ at a heating rate of 15 ℃ min^{-1} and held at 540 ℃ for 120 minutes, followed by cooling down to room temperature at ~10 ℃ min^{-1}.

1.1.1. Synthesis of patterned 3D CMT

To produce the bulk CMT with a patterned surface, 50 mg of 3-TBTBP powder was loaded in a small glass holder with patterned silicon bottom plate. The precursor melted and polymerized in the holder during the heating. After cooling to room temperature, the patterned CMT was peeled off and used directly for testing.

Synthesis of 3D CMT/AAO membrane: the precursor 3-TBTBP was coated on an AAO disc by filtering 3-TBTBP/IPA (4 mg in 50 ml) dispersion through a porous AAO membrane (Anodisc, 47 mm in diameter, 0.02 μm pore size, Whatman). The 3-TBTBP coated AAO disc was covered with a flat Al foil to ensure no precursor evaporated out during the heating. After cooling down to room temperature, the Al foil was easily peeled off and the CMT/AAO film was directly used for tests.

1.1.2. Preparation of 2D CMT on Si Wafer

Prior to the growth of CMT films, O_2 plasma treatment was conducted to remove organic contamination on the Si/SiO_2 wafer. 10 mg of 3-TBTBP was used as precursor

and Si wafer was placed around the precursor source with a certain distance inside the jar. A 10 cm distance results in a CMT film on the wafer with thickness ~ 5 nm.

1.1.3. Preparation of 1D CMT nanotubes

The substrates Cu nanowires were prepared as follows: into 19 ml of $CuCl_2•2H_2O$ aqueous solution (0.01 M) was added 0.12 ml oleylamine. After the mixture was sonicated for 1 min to form a white-blue emulsion, 1 ml L-ascorbic acid (0.2 M) was added and the solution was kept at 60 ℃ overnight. The precipitate was collected by filtration and washed 3 times with isopropanol (IPA). 10 mg of 3-TBTBP was used as the precursor source and 5 mg of Cu nanowire was put near the source (~2 cm in distance) inside the jar. Then the sample was heated to produce CMT coated Cu nanowires. Cu nanowires were etched away by HCl solution (37 wt% in water), then the CMT nanotubes were collected and washed with deionized water, followed by drying at ambient conditions.

1.1.4. Preparation of CMT ultrathin sheets

3-TBTBP and NaCl was mixed evenly and then heated in a jar. When the weight ratio of 3-TBTBP:NaCl = 1:6000, the resulting sheets have a thickness ~ 5.0 nm. After cooling down to room temperature, the CMT coated NaCl was immersed in a large amount of DI water for 3 hours to remove NaCl. The floating CMT sheets were filtered and washed with DI water. Finally, the obtained CMT sheets were freeze-dried.

2. Structural characterization of 3-TBTBP and CMT

2.1.Methods and instruments

Atomic Force Microscope (AFM) measurements were operated with a Bruker Dimension Fast Scan Atomic Force Microscope in tapping mode at ambient conditions. Scanning electronic microscopy (SEM) images were obtained using a Jeol JSM-6701F instrument. For X-ray photoelectron spectroscopy (XPS) investigations, samples were dispersed on Au-coated Si wafers. The XPS spectra were collected with the Phobios 100 electron analyzer equipped with 5 channeltrons, using an unmonochromated Al Ka X-ray source (1486.6 eV). Fourier-transform infrared spectroscopy (FTIR) measurements were performed at room temperature in a continuous vacuum environment. The FTIR samples were prepared by compressing samples with KBr into a disc. Solid ^{13}C NMR spectra were recorded with Bruker 400 MHz NMR. UV-VIS-NIR adsorption spectra were collected with Shimadzu UV-3600. The samples were dispersed in DMF. Bulk CMT was ground into finepowders for Ar sorption measurements, which were performed on a Micromeritics ASAP 2020 with micropore option using liquid Ar bath. Thermal gravimetric analysis (TGA) was performed in the range of 150 to 900 ℃ at a heating rate of 10 ℃ /min in nitrogen atmosphere. Differential scanning calorimetry (DSC) was measured in the range of 100 to 600 ℃ at a heating rate of 15 ℃ min^{-1} in nitrogen atmosphere.

The free volume size, fraction and distribution of CMT polymer powders as a function of temperature were characterized by positron annihilation lifetime spectroscopy (PALS) using a conventional fast-fast coincidence spectroscope. Two Kapton® films were used to sandwich a radioactive source of ^{22}Na as the source of positrons. The samples were vacuumed for at least 12 h prior to testing and five million counts were collected for each spectrum. The data were resolved into three lifetimes using the PATFIT program, which assumes a Gaussian distribution of the logarithm of the

lifetime for each component. The free volume distribution was analysed based on the MELT program.

Density of CMT was determined using a balance (Mettler Toledo, XS205) and a density determination kit according to the Archimedes' principle. The CMT density (ρ_c) was calculated based on the following equation:

$$\rho_c = \frac{W_a}{W_l}\rho_l \qquad \text{(S1)}$$

where W_a is the CMT weight in air, W_l is the CMT weight in the auxiliary liquid and ρ_l is the density of the auxiliary liquid (*n*-hexane).

2.2. Crystallography data collection

The X-ray data were collected with a Bruker AXS D8 Venture Kappa four cycles X-ray diffractometer system equipped with a Photon 100 detector, using a Mo sealed microfocusing source, with the Bruker Apex 2 suite program. Data were integrated with the Bruker SAINT program using a narrow-frame algorithm. SADABS was used for absorption correction. Structural solution and refinement were carried out with the SHELXTL suite of programs. The structures were solved by direct methods, followed by difference maps and refined with full-matrix least-squares on F2. All non-hydrogen atoms were generally given anisotropic displacement parameters in the final model. All hydrogen atoms were placed at calculated positions.

2.3. Crystal structure of 3-TBTBP

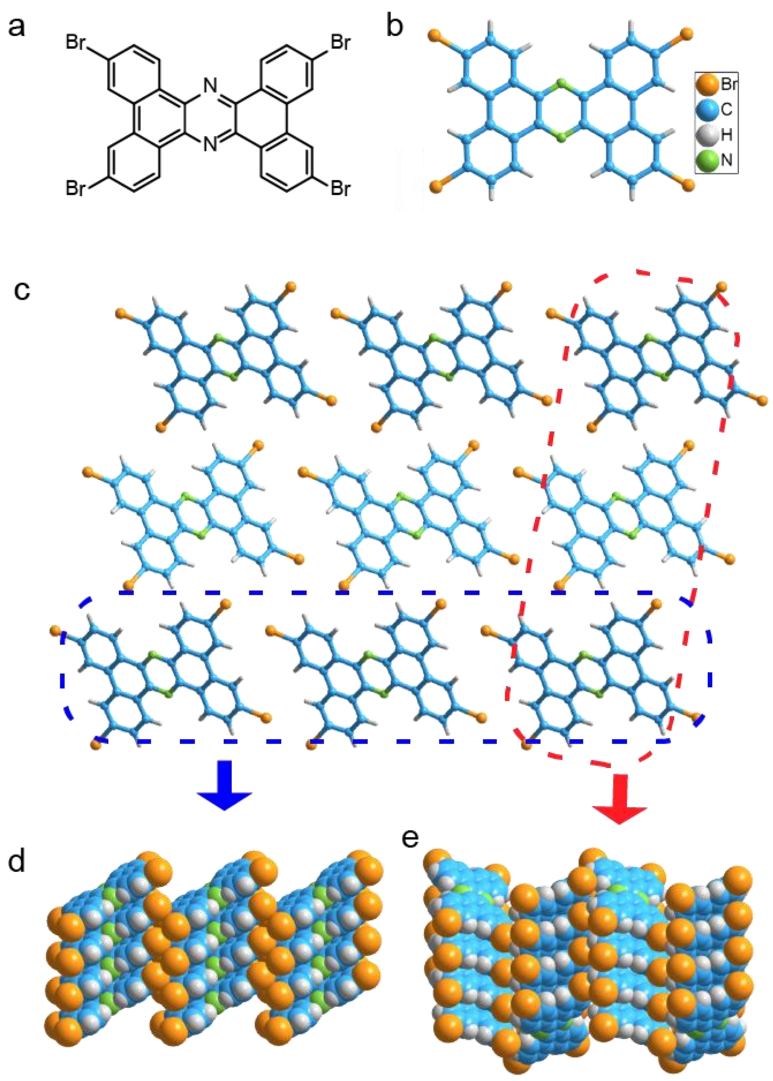

Supplementary Figure 1 | ** Crystal structure of 3-TBTBP**. **a**, Chemical structure of 3-TBTBP. **b**, Molecular structure in ball-stick modes. **c**, Top view along *b*-axis. **d**, Parallel-stacked view along *c*-axis. **e**, Non-parallel-stacked view along *a*-axis. 3-TBTBP crystals are generated by crystallographic inversion center at the center of the 3-TBTBP molecules. The adjacent molecules consist of parallel and non-parallel stacked alignments along *c*-axis and *a*-axis, respectively. 3-TBTBP molecules induce large face-to-face π-π interactions with an intermolecular distance of 3.434 Å. The stacked structure is stabilized by supramolecular multiple interactions such as H-bonds, face-to-face π-π interactions, and van der Waals forces.

2.4. X-ray photoelectron spectroscopy (XPS) spectra of 3-TBTBP and CMT

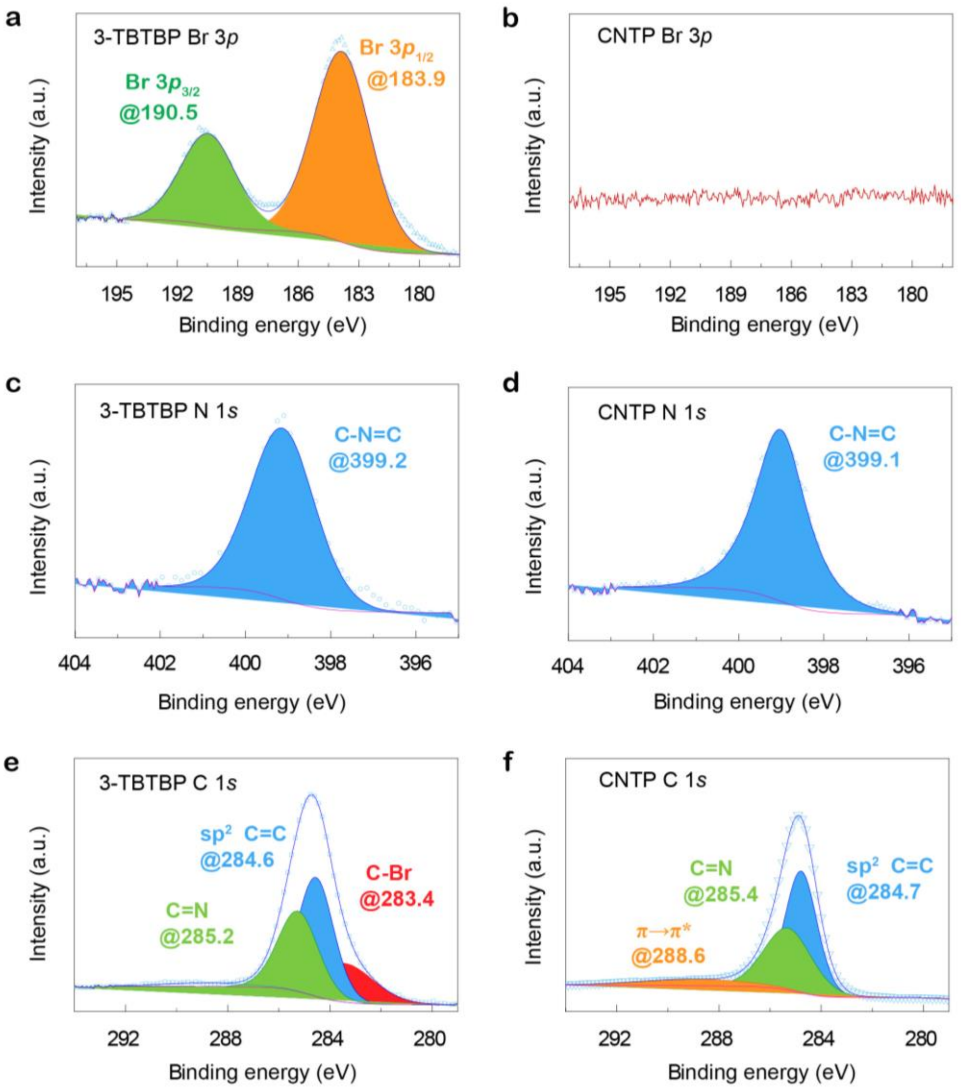

Supplementary Figure 2 | X-ray photoelectron spectroscopy (XPS) spectra of 3-TBTBP and CMT. **a**, **c**, and **e**, Br 3*p*, N 1*s* and C 1*s* of 3-TBTBP, respectively. **b**, **d** and **f**, Br 3*p*, N 1*s* and C 1*s* of CMT, respectively. The Br peaks of aryl–Br (Br $3p_{3/2}$ at 190.5 eV and Br $3p_{1/2}$ at 183.9 eV) largely disappeared, indicating debromination, while the N peak of C–N=C (at ~399.2 eV) remained after polymerization.

2.5. FTIR spectra of 3-TBTBP and CMT

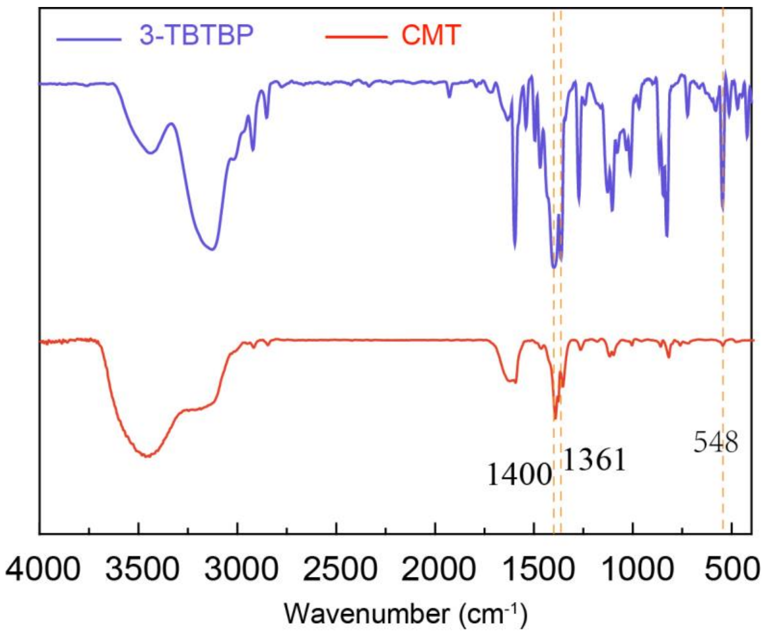

Supplementary Figure 3 | FTIR spectra of 3-TBTBP and CMT. The absorption band at 548 cm^{-1} corresponds to the Ar–Br vibration mode, which largely disappears after polymerization.

2.6. Solid state ^{13}C NMR spectroscopy of 3-TBTBP and CMT

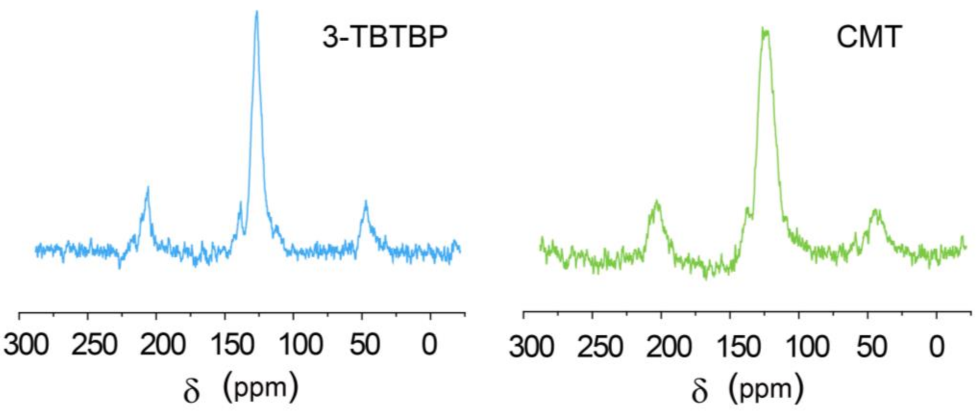

**Supplementary Figure 4 | ** Solid state ^{13}C NMR of 3-TBTBP and CMT

2.7. Elemental analyses of 3-TBTBP and CMT

**Supplementary Table 1 | ** Elemental Analyses of 3-TBTBP and CMT

	**3-TBTBP**		**CMT**	
	E[a]	T[b]	E[a]	T[b]
**C** (wt%)	46.58	48.32	83.14	89.38
**H** (wt%)	1.97	1.74	2.58	3.18
**N** (wt%)	4.01	4.02	7.15	7.44
**Br** (wt%)	45.80	45.92	4.38	0
total	98.36[c]	100	97.25[c]	100

[a] Experimental results. [b] Theoretical value. [c] Differences between the experimental results and the theoretical values are likely caused by small molecules trapped in the pores (oxygen, water and etc.).

2.8. SEM images of 3-TBTBP crystals and CMT

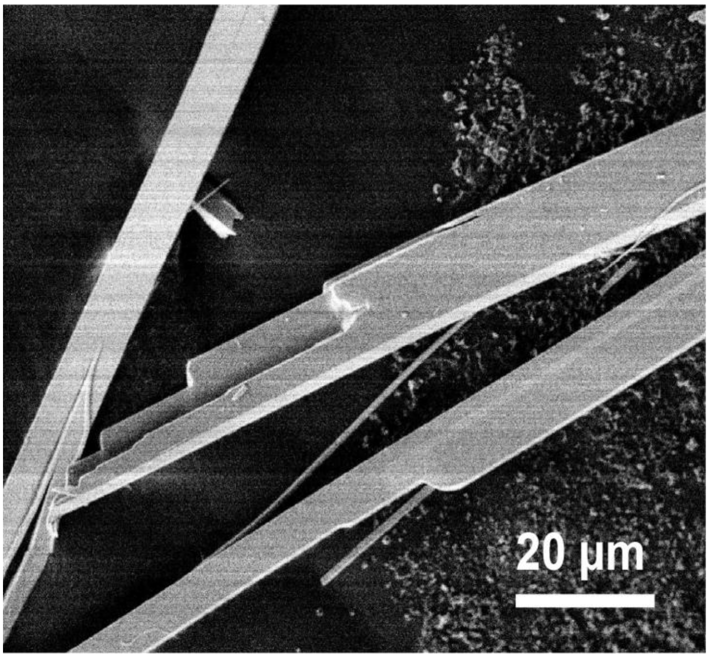

Supplementary Figure 5 | Needle-like 3-TBTBP crystals under SEM. The crystals are prepared by sublimation in a tube furnace, see details in Methods.

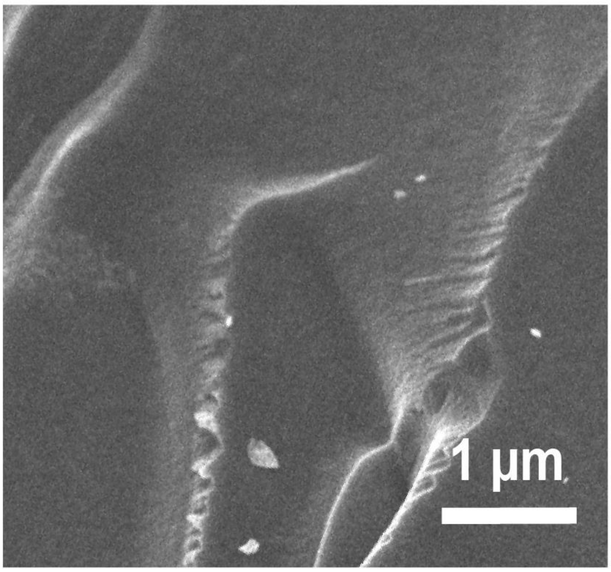

Supplementary Figure 6 | SEM image showing the inner structure of bulk CMT with a dense morphology.

2.9. AFM image of a CMT film grown on Si wafer

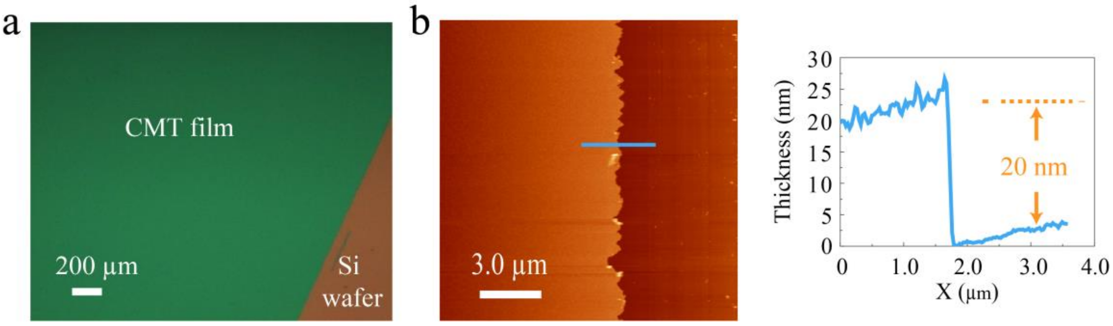

Supplementary Figure 7 | A CMT film grown on Si wafer with a thickness ~20 nm. a, optical microscope image. **b**, AFM topography image and corresponding height profile.

2.10. Raman spectrum of CMT film on Si wafer

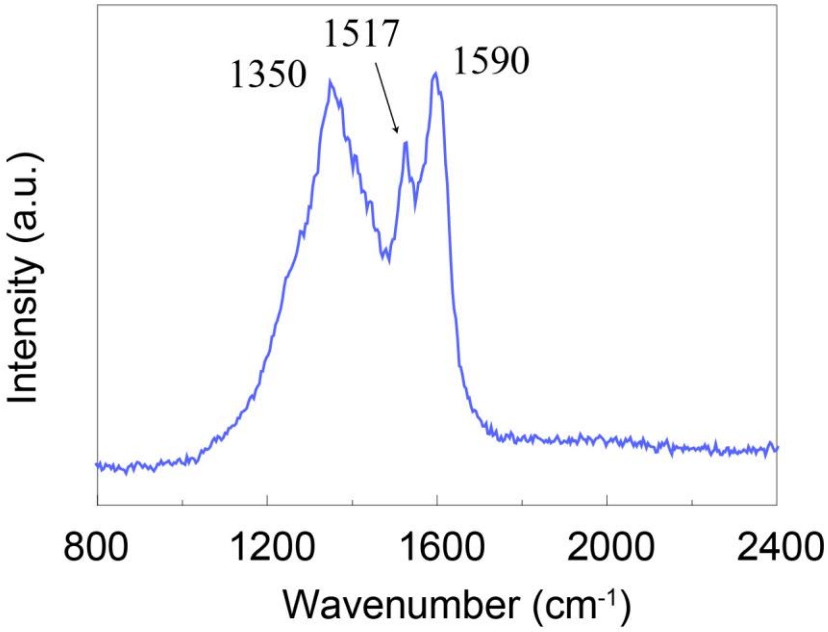

Supplementary Figure 8 | Raman spectrum of CMT film grown on Si wafer.

2.11. SEM image of CMT coated Cu nanowires

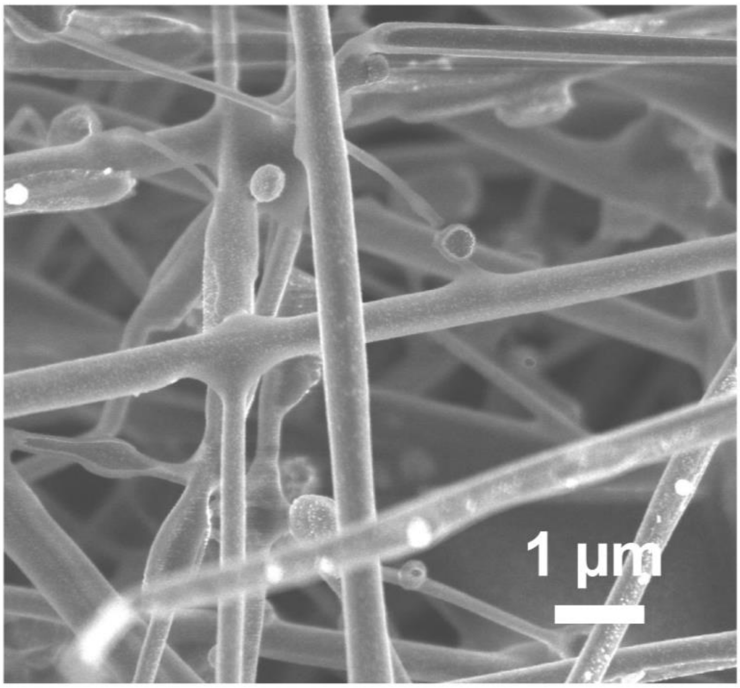

**Supplementary Figure 9 | ** SEM images of CMT coated Cu nanowires.

2.12. Free volume diameter distribution in CMT

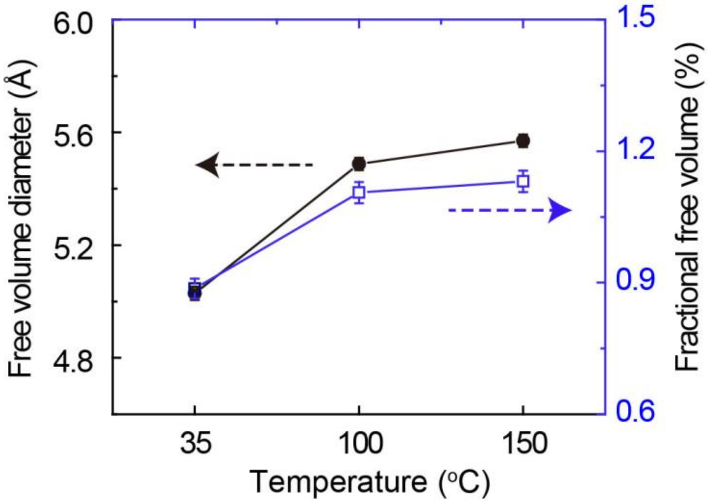

**Supplementary Figure 10 | ** The free volume diameter and fractional free volume of CNTP polymer at different temperatures as characterized by PALS.

2.13. Optical properties of CMT film

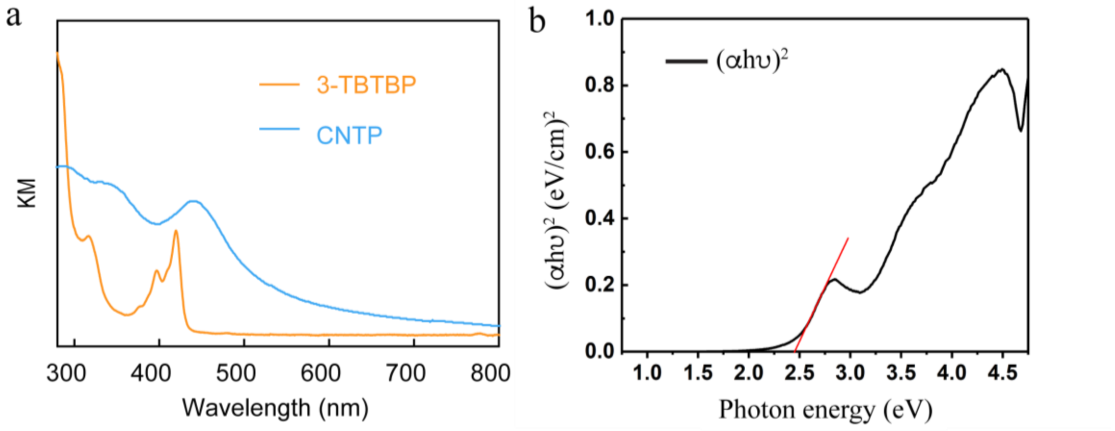

Supplementary Figure 11 | a, UV-Vis absorption of 3-TBTBP and CMT. **b**, Tauc plot calculated based on UV-Vis absorption spectrum of CMT. The Band gap was calculated from the Tauc plot of $(\alpha h\upsilon)^2$ versus $h\upsilon$, where α is the absorption coefficient, h is the Plank constant, and υ is the frequency. The linear part of the Tauc plot was taken and fitted, and the intercept at x-axis was calculated to give a direct band gap of 2.45 eV.

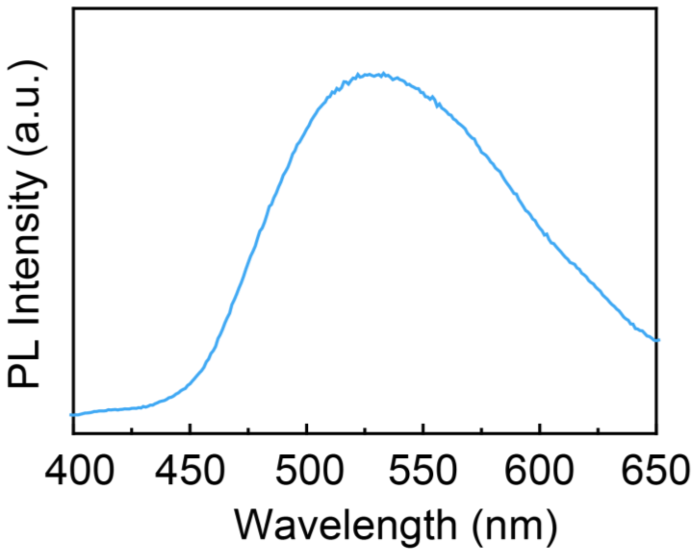

Supplementary Figure 12 | Photoluminescence spectrum of CMT film on Si wafer shows a peak at ~525 nm, suggesting a band gap of ~2.36 eV.

2.14. Optical microscope image of CMT sheets

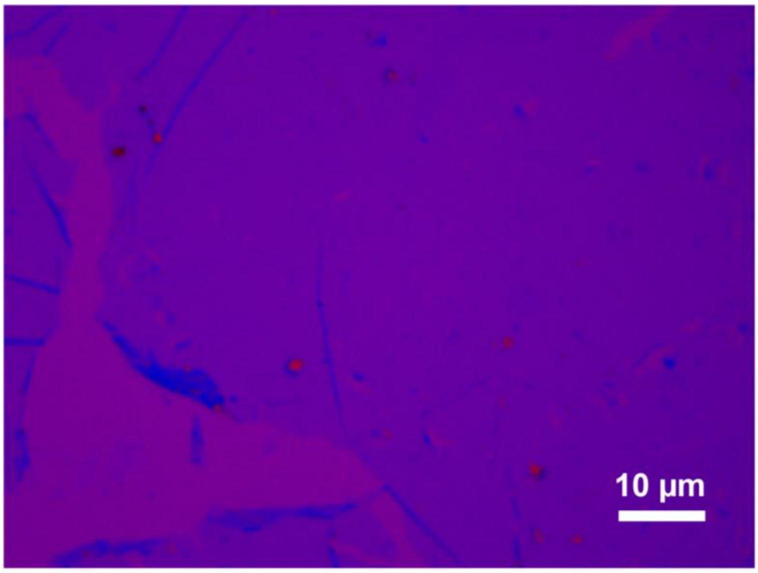

Supplementary Figure 13 | Optical microscope image of ultrathin CMT sheets drop-cast on Si wafer.

2.15. TEM image of CMT sheets

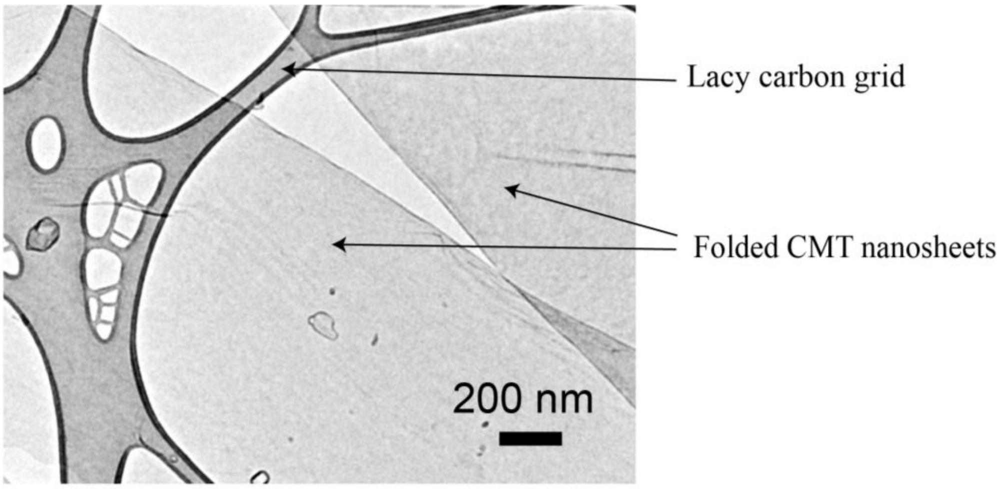

Supplementary Figure 14 | Ultrathin CMT sheets under TEM.

2.16. XRD profile of CMT

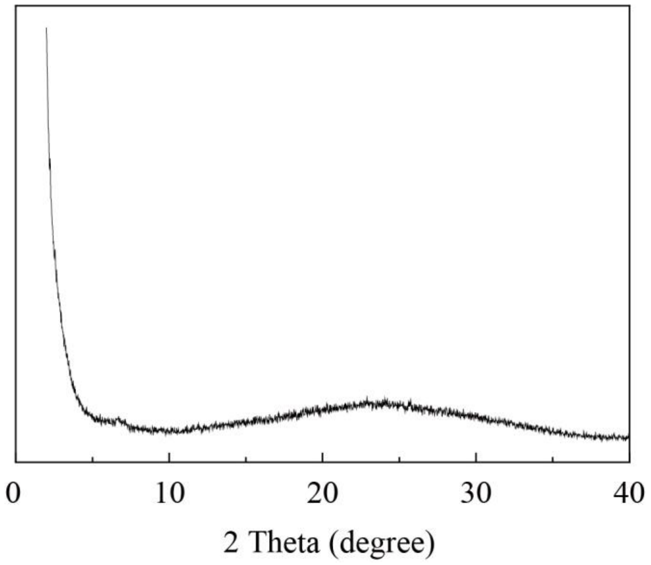

Supplementary Figure 15 | XRD profile of CMT

3. Gas permeation measurements

Gas permeation properties of pure gases were conducted on a constant-volume variable-pressure gas permeation cell. The membrane was mounted onto the permeation cell and vacuumed for at least 12 h before tests. Pure gases including He, H_2, O_2, N_2, CH_4, C_3H_6, C_3H_8 and CO_2 were tested. The measurement condition was held at 30 ℃ with a transmembrane pressure of 1 bar. The gas permeability through the membrane was calculated according to the steady state pressure increment (d*p*/d*t*) as given by the following equation:

$$P = \frac{273\times10^{10}}{760}\frac{Vl}{AT(P_2 \times\frac{76}{14.7})}\frac{dp}{dt} \qquad (1)$$

where P denotes the gas permeability in barrer (1 barrer = 1×10^{-10} cm^3 (STP) cm cm^{-2} s^{-1} $cmHg^{-1}$), V is the volume of the downstream reservoir (cm^3), A is the effective membrane area (cm^2), l represents the membrane thickness (cm), T is the testing temperature (K) and P_2 is the upstream pressure of the system.

To measure the mixed gas separation performance, an equimolar mixture of H_2 and CO_2 was applied as the feed from 30 ℃ to 150 ℃ at a transmembrane pressure of 1 bar. The permeabilities of H_2 and CO_2 were obtained from equations (2) and (3):

$$P_{\mathrm{H_2}} = \frac{273\times10^{10}}{760}\frac{y_{H_2}Vl}{AT(P_2 \times\varphi_{H_2}\times x_{H_2}\times\frac{76}{14.7})}\frac{dp}{dt} \qquad (2)$$

$$P_{\mathrm{CO_2}} = \frac{273\times10^{10}}{760}\frac{y_{CO_2}Vl}{AT(P_2 \times\varphi_{\mathrm{CO_2}}\times x_{\mathrm{CO_2}}\times\frac{76}{14.7})}\frac{dp}{dt} \qquad (3)$$

The molar fractions of gases in the feed and the permeate sides are denoted as x and y, respectively. $\varphi_{\mathrm{H_2}}$ and $\varphi_{\mathrm{CO_2}}$ refer to the fugacity coefficients of the respective upstream gases, which were determined by Thermosolver software according to the Peng-Robinson equation of state[1].

The ideal or mixed gas selectivity (α) between two different gases across a membrane is the ratio of their single gas permeability as described in the following Equation:

$$\alpha_{A/B} = \frac{P_A}{P_B} \tag{4}$$

where P_A and P_B refer to the permeabilities of gases A and B, respectively.

3.1. Gas permeability and ideal selectivity of CMT membranes

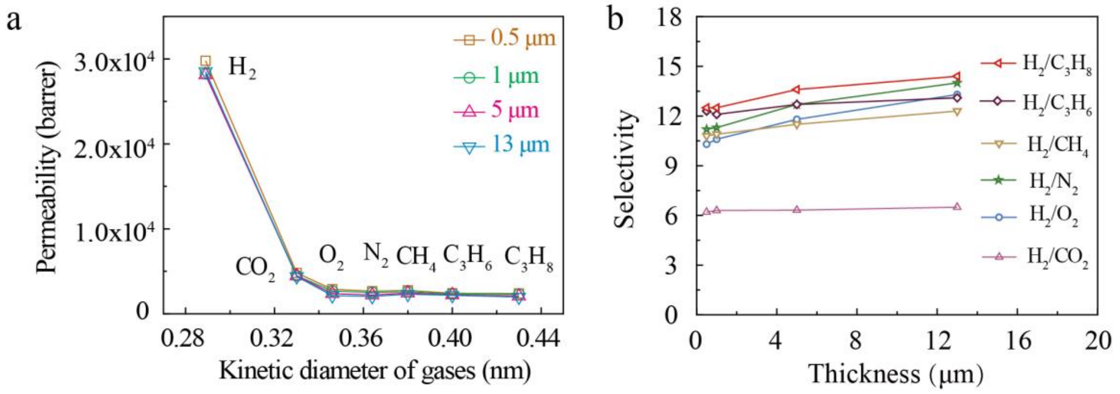

Supplementary Figure 16 | a. Single-gas permeabilities (H_2, CO_2, O_2, N_2, CH_4, C_3H_6 and C_3H_8) through CMT membranes at 30 ℃ and a transmembrane pressure of 1 bar. **b,** The ideal selectivity of CMT membranes with different thickness at 30 ℃ and a transmembrane pressure of 1 bar.

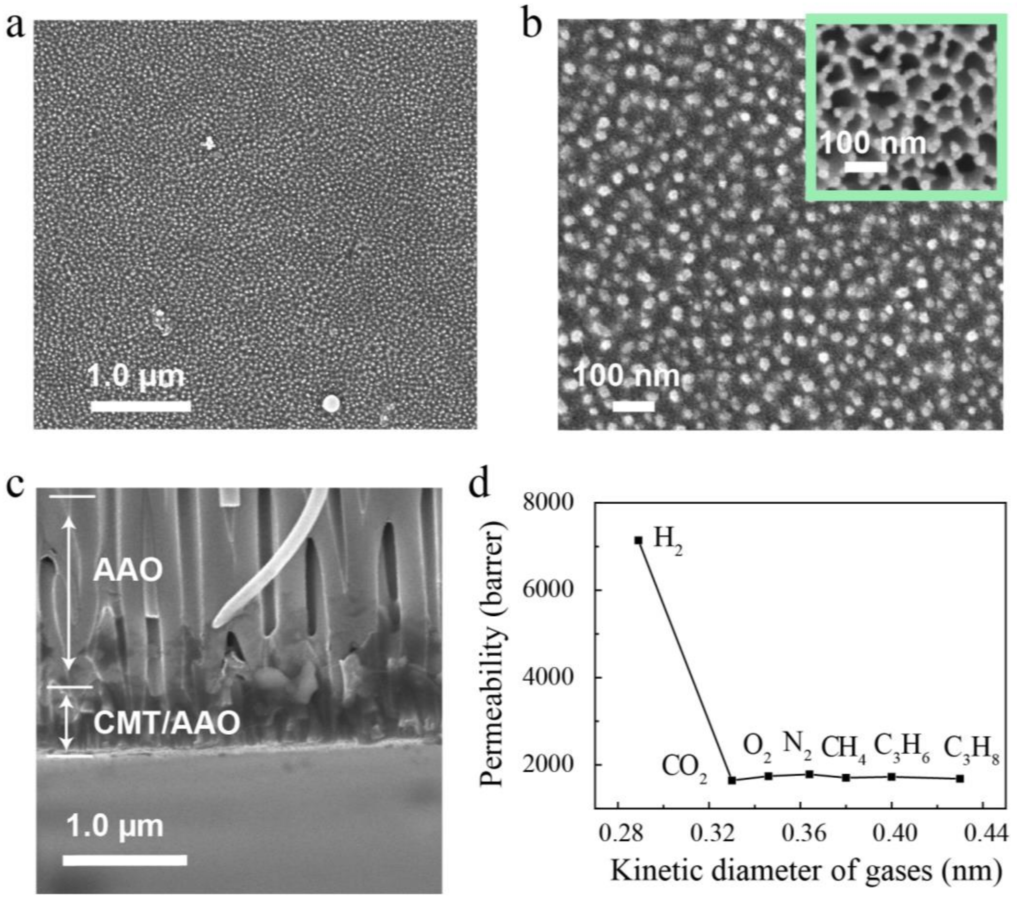

Supplementary Figure 17 | 3D CMT film grown on AAO filter membrane. **a**, SEM image of CMT grown on porous AAO disc (Anodisc, 47 mm in diameter, 0.02 μm pore size, Whatman). **b**, Enlarged SEM image of CMT/AAO membrane. Insert: original uncoated AAO disc. **c**, Cross-sectional SEM image of CMT/AAO, indicating that the thickness of CMT is ~500 nm. **d**, Single-gas permeabilities (H_2, CO_2, O_2, N_2, CH_4, C_3H_6 and C_3H_8) through the 3D CMT/AAO membrane at 30 ℃ and a transmembrane pressure of 1 bar.

3.2. Mixed H_2/CO_2 gas separation performance and CO_2 sorption isotherms of CMT membrane

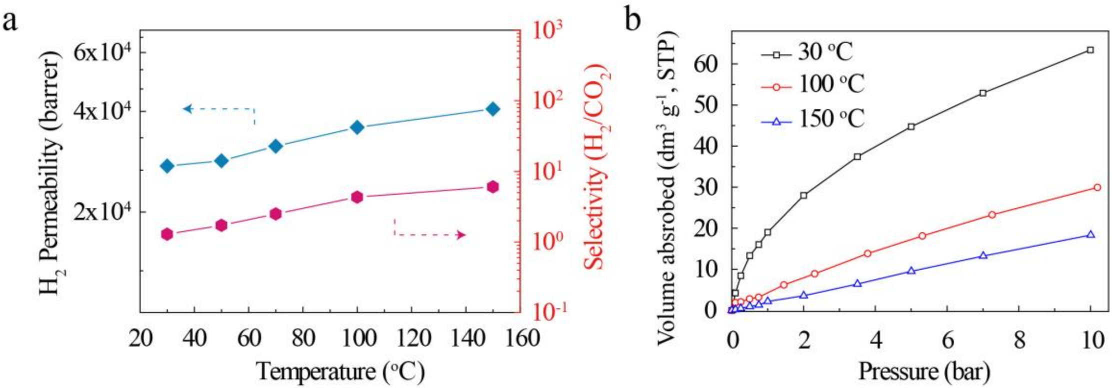

Supplementary Figure 18 | a, Mixed H_2/CO_2 permeability and selectivity of a 1 μm-thick CMT membrane as a function of temperature in equimolar mixed-gas permeation tests. **b**, CO_2 sorption isotherms of CMT at different temperatures. Adsorbed CO_2 decreases as a function of increasing temperature. It shows that the concentration of adsorbed CO_2 inside the membranes decreases as a function of increasing temperature owing to the larger thermal energy of CO_2 at higher temperatures. Due to the decrease of CO_2 adsorbed concentration, the solubility coefficient decreased (S=C/P, C refers to the concentration of adsorbed CO_2, P refers to the applied pressure), thereby reducing the permeability of CO_2. Therefore, the H_2 permeability is increased together with the increase of H_2/CO_2 selectivity.[2]

3.3. Comparison of gas separation performance of CMT with various reported membranes

**Supplementary Table 2 | ** Comparative pure gas permeabilities and selectivities for CMT and accumulated literature data.

No. of membrane materials indicated in Figure 3b-d		Permeability (barrer)				Ideal selectivity			Ref.
		H_2	CO_2	N_2	CH_4	H_2/CO_2	H_2/N_2	H_2/CH_4	
CMT		29786	4807	2659	2753	6.2	11.2	10.8	This work
1	PIM-TMN-Trip-1	16900	33300	2230	3420	0.5	7.6	4.9	3
2	PIM-BTrip	12100	21500	1190	1690	0.6	10.2	7.2	4
3	PIM-BTrip-TB	9980	13200	926	1440	0.7	10.7	6.9	5
4	PIM-C1	9870	18900	980	1310	0.5	10.1	7.5	6
5	PIM-SBF-2	9160	22300	1150	2020	0.4	7.9	4.5	7
6	PIM-Trip-TB	8039	9709	629	905	0.8	12.7	8.8	8
7	PIM-EA-TB	7760	7140	525	699	1.1	14.7	11.1	9
8	PIM-TMN-SBI	7190	17500	1080	2100	0.4	6.6	3.4	3
9	PIM-TMN-Trip-TB	6100	6060	396	710	1.0	15.4	8.5	3
10	PIM-SBF	6320	13900	786	1100	0.4	8.0	5.7	10
11	PIM-1	5010	13600	823	1360	0.3	6.1	3.6	10
12	EAD-DMN-172	4703	8070	480	707	0.5	9.8	6.6	11
13	KAUST-PI-1	3983	2389	107	105	1.6	37.2	37.9	12
14	TPIM	2666	1549	54	50	1.7	49.3	53.3	13
15	TPBO	1701	1433	70	40	1.2	24	43	14
16	SIM-1	6123	2606	2464	2471	2.3	2.4	2.4	15
17	ZIF-8	3566	615	375	321	5.7	9.5	11.1	16
18	MXene	2402.3	11	20	3.1	218.3	120.1	774.9	17

19	MFI zeolite (723K)	2370	16.8	37.6	13.1	141	63	180	18
20	CAU-1	2310	175	240.6	213.8	13.2	9.6	10.8	19
21	MoS_2	1175	142	180	200	8.29	6.5	5.8	20
22	COF-LZU1-ACOF	732	27	8	7	26.7	88.7	105	21
23	P33DT-ThC4	614	13	8.5	9.1	47	73.6	68.8	22
24	GO	513	1.9	2.2	4.7	270	233.1	109.1	23'
25	2D-ZIF	326	3.2	8.1	4.6	101.8	40.2	70.8	24
26	ZIF-7	272	21	13	19	13	21	15	25

4. Molecular dynamics simulations

4.1. Construction and validation of CMT model.

The amorphous 3D CMT membranes were constructed by adopting Polymatic, which is a simulated polymerization algorithm proposed by Colina.[26] During the simulation, non-polarizable all-atom OPLS-AA force field[27] was used for all structures. Prior to polymerization, 160 monomers excluding bromine atoms (Br) were inserted randomly in a periodic cubic cell with dimensions of $50 \times 50 \times 50$ Å^3. Then the polymerizations of monomer precursors to CMT were performed via multiple cycles including bonding at high temperature (813 K) and equilibrium at targeted temperature of 303 K (or 423 K). When no bonding occurred, about 160 bonds were formed in these two systems (303 K and 423 K). Then the removed Br was added back to these sites without bonding. The equilibrium structures were obtained by annealing, as shown in Supplementary Figure 18. All the simulations were complemented with LAMMPS package[28].

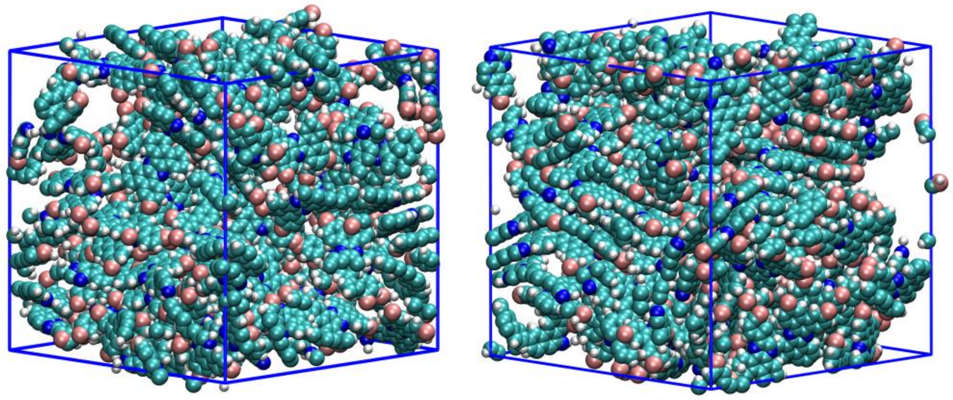

**Supplementary Figure 19 | ** The 3D CMT models built at 303K (left) and 423K (right). Color code: C, cyan; N, blue; H, white; Br, pink.

In order to validate the reasonability of the built CMT model, the pore size distributions (Supplementary Figure 19) of CMT models were obtained by sampling the test particle radii proposed by Bhattacharya et al[29]. The difference between the simulated and experimental pore size distribution could be considered as favorable from the simulation method.[30] Furthermore, both mass density and accessible surface area were tested to compare with experimental data.

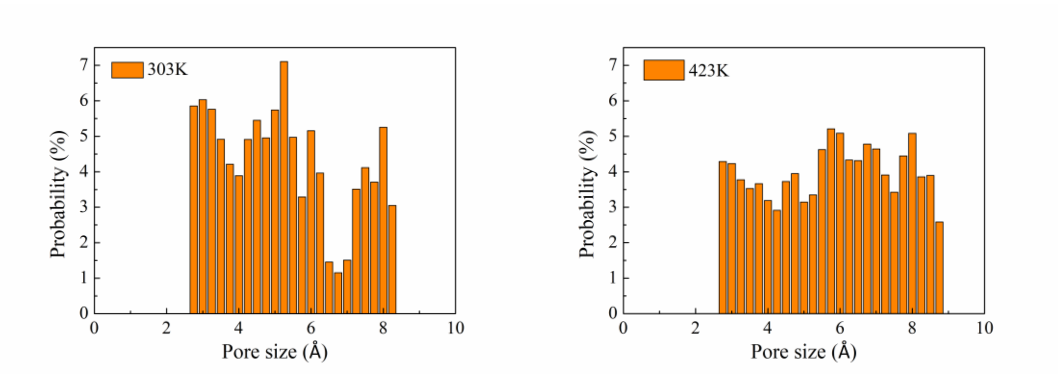

Supplementary Figure 20 | The pore size distribution of CMT obtained at 303 K (left) and 423 K (right).

Supplementary Table 3 | The density and accessible surface area tested in simulation and experiment.

	Density (g cm^{-3})	Accessible surface area probed by Ar gas (m^2 g^{-1})
Simulation	1.14	911
Experiment	1.17	838

4.2. MD simulation of gas separation

4.2.1 Gas separation model construction

A typical bichamber model (Supplementary Figure 20) with dimensions of 50 Å ×50 Å ×580 Å was constructed to test the H_2/CO_2 separation performance. A 5 nm thick CMT layer obtained at 423 K, which is the single flake thickness of laminated CMT membrane used in experiment, was employed as the separation membrane in the simulation. An equimolar H_2/CO_2 gas mixture with a total number of 600 molecules was placed into the left chamber, maintaining initial pressure of 5 MPa. A 250 Å vacuum chamber was placed on the other side as permeate chamber. Periodic boundary conditions were used in all three directions. Two rigid graphene sheets were placed at the rightmost and leftmost sides of the system to prevent gas diffusion between the feed and permeate chambers.

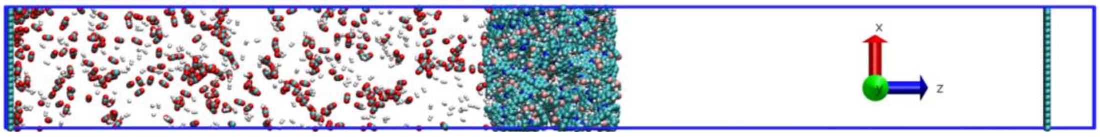

Supplementary Figure 21 | Gas separation model composed of feed (left) and permeate (right) chambers are adopted to separate binary H_2/CO_2 by a 5 nm-thick CMT membrane. Color codes: White, red, cyan, blue, and pink balls denote hydrogen, oxygen, carbon, nitrogen and bromine, respectively.

4.2.2 MD simulation of gas separation process

For H_2 and CO_2, three-site models were adopted[31]. The 12-6 Lennard-Jones potential was used to calculate the van der Waals interactions between gas and CMT membrane. Partial atomic charges and L-J parameters of H_2 (CO_2) and CMT are listed in Supplementary Table 4. Lorentz-Berthelot mixing rules were used to obtain the

missing heteronuclear parameters. The cutoff distance for all the short-range vdW interactions was set as 12 Å. The long-range electrostatic interactions were computed by using the particle-particle particle-mesh (PPPM) algorithm. The simulation was conducted in a canonical ensemble (NVT) at 423 K controlled by the Nose-Hoover thermostat method[32]. The time step was set as 1 fs and the data were collected every 1 ps. The total simulation time of each model was 20 ns. The simulation shows that he fluxes are 88.9 and 22.4 molecules/ns for H_2 and CO_2, respectively, giving a H_2/CO_2 selectivity ~4 for the 5 nm-thick monolayer CMT sheet.

Supplementary Table 4 | Partial atomic charges and Lennard-Jones parameters for gas molecules and CMT

	CMT				CO_2		H_2	
	C	N	H	Br	C	O	H	Center of Mass
ε (kcal/mol)	0.070	0.170	0.030	0.470	0.0559	0.160	0	0.0729
σ (Å)	3.550	3.250	2.420	3.470	2.757	2.565	0	2.958
q / |e|	assigned by OPLS-AA forcefield				0.6512	-0.3256	0.4680	-0.9360

5. Crystallographic data of 3-TBTBP

Supplementary Table 5 | Crystallographic data of 3-TBTBP

**Chemical formula**	$C_{28}H_{12}Br_4N_2$	
**Formula weight**	696.04 g mol^{-1}	
**Temperature**	100(2) K	
**Wavelength**	0.71073 Å	
**Crystal size**	0.090 × 0.274 × 0.574 mm	
**Crystal system**	monoclinic	
**Space group**	P 1 2(1)/c 1	
**Unit cell dimensions**	a = 13.2374(8) Å	α = 90°
	b = 4.3613(3) Å	β = 101.649(3)°
	c = 19.8717(11) Å	γ = 90°
**Volume**	1123.61(12) Å^3	
**Z**	2	
**Density (calculated)**	2.057 g cm^{-3}	
**Absorption coefficient**	7.186 mm^{-1}	
**F(000)**	668	
**Theta range for data collection**	2.86 to 26.36 °	
**Index ranges**	-16<=h<=15, -5<=k<=5, -24<=l<=16	
**Reflections collected**	4159	
**Independent reflections**	2276 [R(int) = 0.0565]	
**Coverage of independent reflections**	98.6%	
**Absorption correction**	multi-scan	
**Max. and min. transmission**	0.5238 and 0.0716	

**Refinement method**	Full-matrix least-squares on F^2
**Refinement program**	SHELXL-2014/7 (Sheldrick, 2014)
**Function minimized**	$\Sigma\, w(F_o^2 - F_c^2)^2$
**Data / restraints / parameters**	2276 / 0 / 154
**Goodness-of-fit on F^2**	1.005
**Final R indices**	1811 data; $I>2\sigma(I)$: R1 = 0.0585, wR2 = 0.1525
	all data: R1 = 0.0712, wR2 = 0.1652
**Weighting scheme**	$w=1/[\sigma^2(F_o^2)+(0.1061P)^2]$ where $P=(F_o^2+2F_c^2)/3$
**Largest diff. peak and hole**	2.327 and -1.339 eÅ^{-3}
**R.M.S. deviation from mean**	0.243 eÅ^{-3}

6. Supplementary References

1 Peng, D.-Y. & Robinson, D. B. A New Two-Constant Equation of State. *Industrial & Engineering Chemistry Fundamentals* **15**, 59-64 (1976).

2 Japip, S., Liao, K.-S. & Chung, T.-S. Molecularly Tuned Free Volume of Vapor Cross-Linked 6FDA-Durene/ZIF-71 MMMs for H_2/CO_2 Separation at 150 °C. *Advanced Materials* **29**, 1603833 (2017).

3 Rose, I. *et al.* Polymer ultrapermeability from the inefficient packing of 2D chains. *Nature Materials* **16**, 932 (2017).

4 Comesaña-Gándara, B. *et al.* Redefining the Robeson upper bounds for CO_2/CH_4 and CO_2/N_2 separations using a series of ultrapermeable benzotriptycene-based polymers of intrinsic microporosity. *Energy & Environmental Science* **12**, 2733-2740 (2019).

5 Rose, I. *et al.* Highly Permeable Benzotriptycene-Based Polymer of Intrinsic Microporosity. *ACS Macro Letters* **4**, 912-915 (2015).

6 Zhang, J. *et al.* The enhancement of chain rigidity and gas transport performance of polymers of intrinsic microporosity via intramolecular locking of the spiro-carbon. *Chemical Communications* **52**, 6553-6556 (2016).

7 Bezzu, C. G. *et al.* The synthesis, chain-packing simulation and long-term gas permeability of highly selective spirobifluorene-based polymers of intrinsic microporosity. *Journal of Materials Chemistry A* **6**, 10507-10514 (2018).

8 Carta, M. *et al.* Triptycene Induced Enhancement of Membrane Gas Selectivity for Microporous Tröger's Base Polymers. *Advanced Materials* **26**, 3526-3531 (2014).

9 Carta, M. *et al.* An Efficient Polymer Molecular Sieve for Membrane Gas Separations. *Science* **339**, 303-307 (2013).

10 Bezzu, C. G. *et al.* A Spirobifluorene-Based Polymer of Intrinsic Microporosity with Improved Performance for Gas Separation. *Advanced Materials* **24**, 5930-5933 (2012).

11 Ma, X. & Pinnau, I. Effect of Film Thickness and Physical Aging on "Intrinsic" Gas Permeation Properties of Microporous Ethanoanthracene-Based Polyimides. *Macromolecules* **51**, 1069-1076 (2018).

12 Swaidan, R., Al-Saeedi, M., Ghanem, B., Litwiller, E. & Pinnau, I. Rational Design of Intrinsically Ultramicroporous Polyimides Containing Bridgehead-Substituted Triptycene for Highly Selective and Permeable Gas Separation Membranes. *Macromolecules* **47**, 5104-5114 (2014).

13 Ghanem, B. S., Swaidan, R., Ma, X., Litwiller, E. & Pinnau, I. Energy-Efficient Hydrogen Separation by AB-Type Ladder-Polymer Molecular Sieves. *Advanced Materials* **26**, 6696-6700 (2014).

14 Luo, S. *et al.* Highly Selective and Permeable Microporous Polymer Membranes for Hydrogen Purification and CO2 Removal from Natural Gas. *Chemistry of Materials* **30**, 5322-5332 (2018).

15 Aguado, S. *et al.* Facile synthesis of an ultramicroporous MOF tubular membrane with selectivity towards CO_2. *New Journal of Chemistry* **35**, 41-44 (2011).

16 Zhang, X. *et al.* New Membrane Architecture with High Performance: ZIF-8 Membrane Supported on Vertically Aligned ZnO Nanorods for Gas Permeation and Separation. *Chemistry of Materials* **26**, 1975-1981 (2014).

17 Ding, L. *et al.* MXene molecular sieving membranes for highly efficient gas separation. *Nature Communications* **9**, 155 (2018).

18 Tang, Z., Dong, J. & Nenoff, T. M. Internal Surface Modification of MFI-Type Zeolite Membranes for High Selectivity and High Flux for Hydrogen. *Langmuir* **25**, 4848-4852 (2009).

19 Zhou, S. *et al.* Development of hydrogen-selective CAU-1 MOF membranes for hydrogen purification by 'dual-metal-source' approach. *International Journal of Hydrogen Energy* **38**, 5338-5347 (2013).

20 Achari, A., S, S. & Eswaramoorthy, M. High performance MoS2 membranes: effects of thermally driven phase transition on CO2 separation efficiency. *Energy & Environmental Science* **9**, 1224-1228 (2016).

21 Fan, H. *et al.* Covalent Organic Framework–Covalent Organic Framework Bilayer Membranes for Highly Selective Gas Separation. *Journal of the American Chemical Society* **140**, 10094-10098 (2018).
22 Zhang, M. *et al.* Electropolymerization of Molecular-Sieving Polythiophene Membranes for H_2 Separation. *Angewandte Chemie International Edition* **58**, 8768-8772 (2019).
23 Li, H. *et al.* Ultrathin, Molecular-Sieving Graphene Oxide Membranes for Selective Hydrogen Separation. *Science* **342**, 95-98 (2013).
24 Peng, Y. *et al.* Metal-organic framework nanosheets as building blocks for molecular sieving membranes. *Science* **346**, 1356-1359 (2014).
25 Li, Y., Liang, F., Bux, H., Yang, W. & Caro, J. Zeolitic imidazolate framework ZIF-7 based molecular sieve membrane for hydrogen separation. *Journal of Membrane Science* **354**, 48-54 (2010).
26 Abbott, L. J., Hart, K. E. & Colina, C. M. Polymatic: a generalized simulated polymerization algorithm for amorphous polymers. *Theoretical Chemistry Accounts* **132**, 1334 (2013).
27 Jorgensen, W. L., Maxwell, D. S. & Tirado-Rives, J. Development and testing of the OPLS all-atom force field on conformational energetics and properties of organic liquids. *Journal of the American Chemical Society* **118**, 11225-11236 (1996).
28 Plimpton, S. Fast parallel algorithms for short-range molecular dynamics. *Journal of computational physics* **117**, 1-19 (1995).
29 Bhattacharya, S. & Gubbins, K. E. Fast method for computing pore size distributions of model materials. *Langmuir* **22**, 7726-7731 (2006).
30 Liang, B. *et al.* Microporous membranes comprising conjugated polymers with rigid backbones enable ultrafast organic-solvent nanofiltration. *Nature Chemistry* **10**, 961-967 (2018).
31 Jiao, S. & Xu, Z. Selective gas diffusion in graphene oxides membranes: a molecular dynamics simulations study. *ACS applied materials & interfaces* **7**, 9052-9059 (2015).
32 Nosé, S. A unified formulation of the constant temperature molecular dynamics methods. *The Journal of chemical physics* **81**, 511-519 (1984).
